# Significance of collateral circulation in managing persistent sciatic artery: Two case reports

**DOI:** 10.3389/fsurg.2023.1159463

**Published:** 2023-04-17

**Authors:** Woo-Sung Yun, Hyeon Ju Kim, Deokbi Hwang, Hyung-Kee Kim

**Affiliations:** ^1^ Division of Vascular and Endovascular Surgery, Department of Surgery, Kyungpook National University Hospital, Daegu, Republic of Korea; ^2^ Division of Vascular and Endovascular Surgery, Department of Surgery, Kyungpook National University Chilgok Hospital, School of Medicine, Kyungpook National University, Daegu, Republic of Korea

**Keywords:** embolism and thrombosis, persistent sciatic artery, collateral circulation, embolectomy, conservative treatment

## Abstract

Persistent sciatic artery (PSA) is a rare congenital anomaly considered an embryologic remnant of the internal iliac artery. Traditionally, the classification systems categorized PSA based on the completeness of PSA and superficial femoral artery (SFA) alongside the origin of PSA. The most common class has been known as type 2a in Pillet–Gauffre classification, meaning complete PSA with incomplete SFA. The mainstay of these patients with limb ischemia has been surgical bypass alongside excision or ligation of PSA aneurysm if present. However, the current PSA classification system does not account for collateral blood flow. Herein, we described two cases of type 2a PSA with distal embolization and explored therapeutic choices for PSA based on collateral presence. The first patient was treated with thromboembolectomy and patch angioplasty, and the second with conservative management. Despite distal embolization in both patients, bypass surgery was avoided, and distal circulation was maintained *via* collaterals from deep and superficial femoral arteries without increased risk of recurrent embolization. Thus, carefully examining collateral circulation and customized strategy is essential for managing PSA.

## Introduction

Persistent sciatic artery (PSA) is a rare congenital anomaly that is considered an embryologic remnant of the internal iliac artery (IIA) and has an incidence of 0.03%–0.06% (1). Although most patients are asymptomatic, they can be symptomatic and present with sciatica neuralgia, aneurysm formation, thrombotic occlusion, and distal embolization. The reported rates of complications include an aneurysm in 48% of cases, a stenosis in 7%, an occlusion of the PSA in 9%, and an occlusion of an artery distal to the PSA in 6% (1). Among the symptoms, aneurysm rupture and acute limb ischemia due to thrombosis or distal embolization constitute terrible complications of PSA owing to possible death or limb loss (2, 3).

The treatment of PSA should be tailored to the symptomatology of patients and the classification of PSA (Table 1). Historically, the most prevalent PSA in Pillet–Gauffre class has been known to be a type 2a PSA. The mainstay of patient care in a type 2a PSA and limb ischemia has been surgical bypass in conjunction with PSA aneurysm (PSAA) excision or ligation if present (4). Recently, hybrid treatment and endovascular approaches have emerged as important therapeutic alternatives owing to developments in endovascular techniques and equipment (5). However, the current PSA classification system does not account for collateral blood flow. Moreover, in certain patients with sufficient collaterals, PSA with limb ischemia may be treated with a minimally invasive strategy or conservative approach.

**Table 1:** Comparison of the Ahn–Min and Pillet–Gauffre classifications based on the completeness of the PSA and SFA.

Ahn–Min classification	SFA anatomy	PSA anatomy	PSA aneurysm	Pillet–Gauffre classification
I	Complete	Complete	Absent	Types 1, 5a
Ia			Present	
II	Complete	Incomplete	Absent	Types 3, 4
IIa			Present	
III	Incomplete	Complete	Absent	Types 2a, 2b, 5b
IIIa			Present	
IV	Incomplete	Incomplete	Absent	None
Iva			Present	

PSA, persistent sciatic artery; SFA, superficial femoral artery.

The type 5 lesion in the Pillet–Gauffre classification signifies that the PSA originates from the median sacral artery instead of the internal iliac artery, with either a fully (type 5a) or partially (type 5b) developed SFA. The Ahn–Min classification does not consider the origin of PSA.

Herein, we explored therapeutic choices for PSA based on collateral presence on foot and described two cases of patients with PSA alongside limb ischemia. This study was authorized by the Institutional Review Board (IRB) of our institution (IRB no. 2023-02-004), and the requirement for patient informed consent was waived by IRB.

## Cases

### Case 1

**Figure 1:**
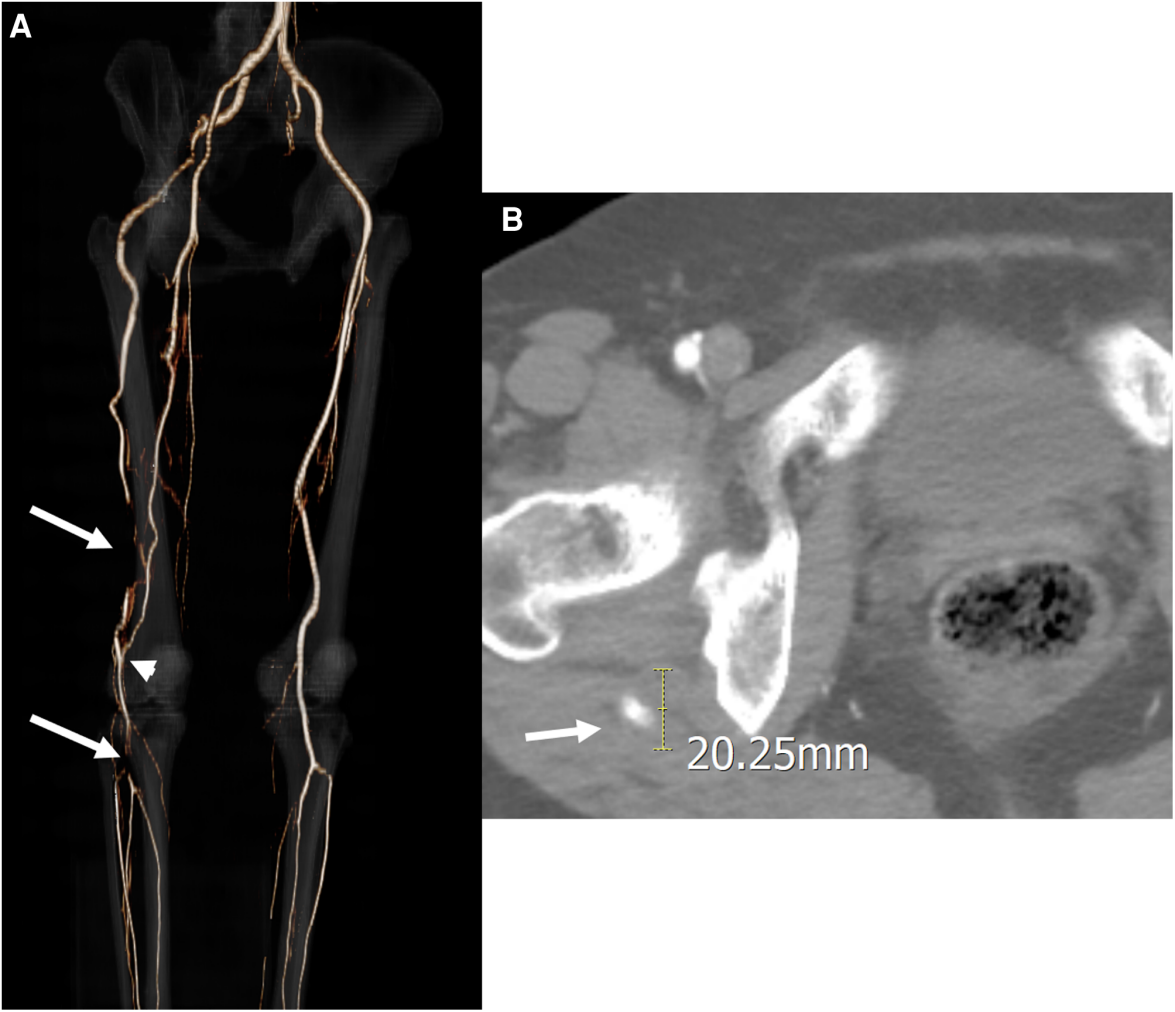
Initial computed tomography angiography (CTA) of the first case. (**A**) Three-dimensional reconstruction image revealed a persistent sciatic artery (PSA) arising from the right internal iliac artery and two distal thromboembolic occlusions located at the distal PSA in the thigh and the popliteal artery at the trifurcation (*arrows*). A collateral arterial flow was detected between the two thromboembolic occlusions (*arrowhead*). (**B**) Axial CTA imaging revealed a PSA aneurysm 20 mm in diameter with a mural thrombus near the posterior part of the greater trochanter of the femur.

A 56-year-old woman presented with a 3-months history of sudden-onset, disabling right leg claudication. She was taking medication for hyperlipidemia and did not have other health problems, like atrial fibrillation or myocardial infarction. Physical examination revealed that, in contrast to the left ankle, the right ankle had no pulse on palpation. The ankle-brachial index (ABI) of the right ankle was 0.62. Computed tomography angiography (CTA) revealed a PSA arising from the right IIA connecting to the above-knee popliteal artery (PA) and a hypoplastic superficial femoral artery (SFA) that was discontinued to the PA (Figure 1A). Additionally, the PSA exposed an aneurysmal change 20 mm in diameter with mural thrombus at the posterior aspect of the greater trochanter of the femur (Figure 1B) alongside two distal thromboembolic occlusions. The proximal thromboembolic occlusion was located in the distal PSA in the distal thigh, and the distal occlusion was located from distal PA to the proximal tibioperoneal trunk. Conversely, a collateral branch of the deep femoral artery (DFA) was joined to the proximal PA between two thromboembolic occlusions.

Our treatment choice focused on the collateral arterial flow between two occlusions. We hypothesized that correcting the distal occlusion at the distal PA would eliminate her symptom, as the collateral flow between the two occlusions could serve as the primary blood supply to the calf and foot. In addition, proximal PSA occlusion at the distal thigh could act as a filter and reduce the risk of further distal embolization.

The distal PA and tibioperoneal trunk were exposed using a proximal calf medial approach. Following 5,000 IU systemic heparinization, a longitudinal arteriotomy was conducted at the occluded segment from the distal PA to the tibioperoneal trunk. Afterward, the old occluding thrombus was extracted using forceps and a Fogarty catheter, and patch angioplasty with the great saphenous vein (GSV) was performed. The GSV was harvested at the ankle, and the single segment of GSV at the thigh and calf was preserved as it was determined that a long bypass would be required if the previously treated segment became occluded again. The proximal thromboembolic occlusion of PSA at the distal thigh was not treated and was left unchanged purposefully in accordance with the aforementioned hypothesis.

**Figure 2:**
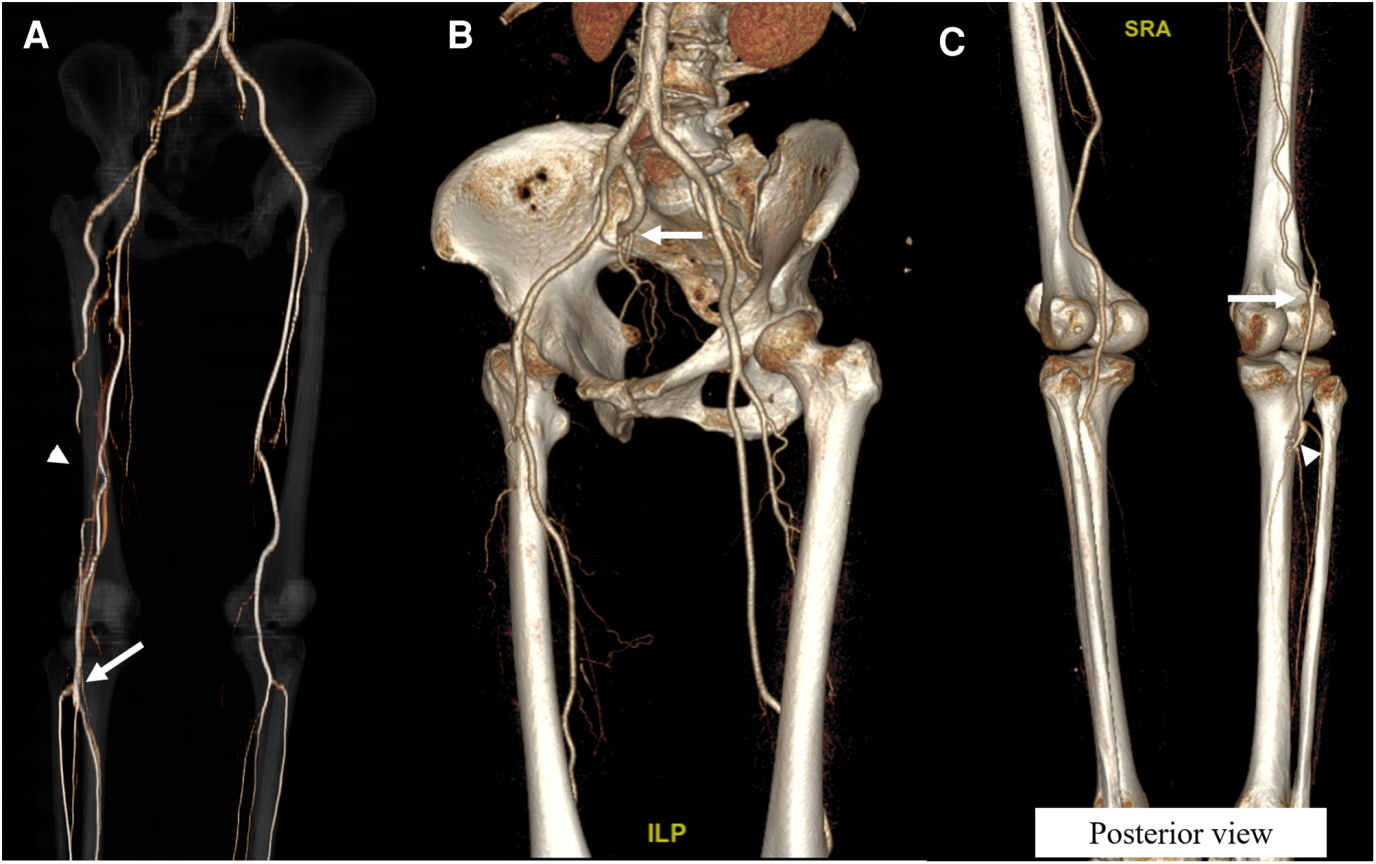
Follow-up computed tomography angiography (CTA) images of the first case. (**A**) Following the operation, a three-dimensional reconstruction image 1 month after the operation demonstrated a well patent distal popliteal artery (*arrow*) and preserved flow from the collaterals from the deep femoral artery (*arrowhead*). (**B**) CTA 3 years after the operation revealed the complete occlusion of the persistent sciatic artery from the buttock (*arrow*). (**C**) Posterior view of the CTA 3 years after the operation demonstrated well patent collaterals from the deep femoral artery (*arrow*) and patent operation site (*arrowhead*).

The patient was discharged 6 days following the operation after a smooth postoperative course. During discharge, the follow-up right ABI improved to 0.85, and the claudication symptom disappeared. The CTA after 1 month of the operation revealed well patent PA supplied by the collateral flow from the DFA, with the PSA remaining occluded at the distal thigh (Figure 2A). Three years after surgery, the CTA revealed the complete occlusion of PSA from the PSAA at the buttock to its connection with the PA and patent operation site (Figures 2B,C). During the 56 months of observation and dual antiplatelet therapy, the patient was asymptomatic.

### Case 2

**Figure 3:**
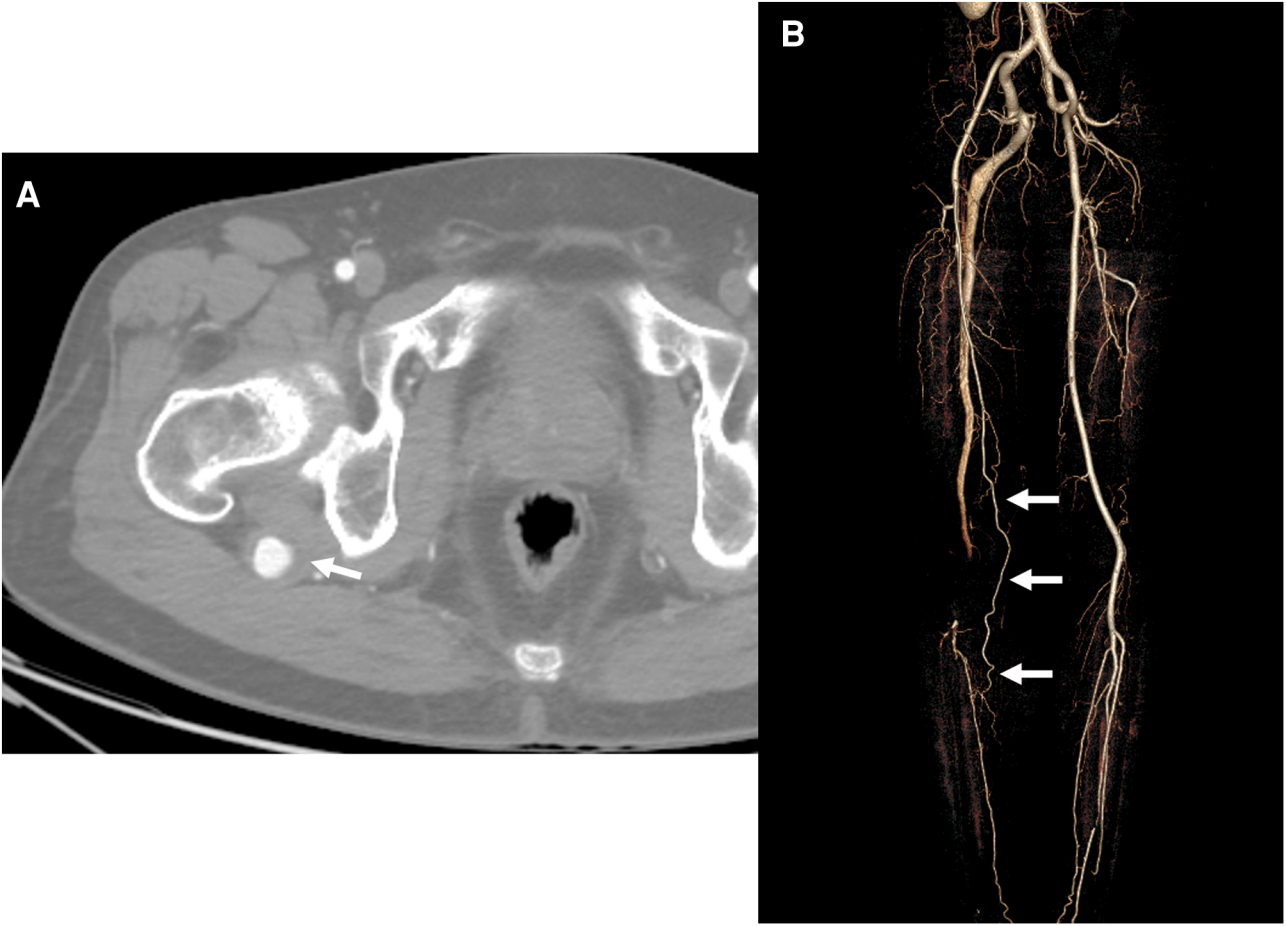
Initial computed tomography angiography (CTA) of the second case. (**A**) Axial image of the CTA revealed an aneurysm with a diameter of 22 m and a mural thrombus of the persistent sciatic artery (PSA) (*arrow*) at the posterior aspect of the greater trochanter of the femur. (**B**) Three-dimensional reconstruction image revealed a collateral flow (arrows) to the calf and foot from the incomplete superficial femoral artery and occluded distal popliteal artery connected with the PSA.

A 63-year-old man presented with sudden onset right foot coldness and claudication for 2 weeks. He had no other medical and surgical comorbidities except radiofrequency ablations for both legs GSV 3 years ago. The electrocardiogram revealed no atrial fibrillation. Physical examination revealed that the left ankle pulse was well palpable, and the right ankle pulse was absent. The ABI on the right ankle decreased to 0.72. The CTA showed a PSA connected to the proximal PA and a 22-mm–sized PSAA with mural thrombus at the posterior aspect of the greater trochanter (Figure 3A). In addition, embolic occlusion was visible in the distal PA. The SFA was incomplete without direct connection to the PA; however, collaterals from the incomplete SFA to the posterior tibial artery (PTA) were identified (Figure 3B).

As the GSVs were unavailable due to prior radiofrequency ablation, we initiated anticoagulation with low-molecular-weight heparin (Clexane®, 1 mg/Kg twice daily) and work-up for revascularization, including echocardiography and quality of other veins for the bypass conduit. Nevertheless, the quality of the arm vein and the small saphenous vein (SSV) was marginal for the bypass graft; the smallest diameter was 2.0 mm on the right SSV, 2.3 mm on the left SSV, 2.1 mm on the right arm vein, and 1.8 mm on the left arm vein. Additionally, throughout the work-up, the claudication symptoms gradually improved. Thus, we selected cilostazol and warfarin for the cautious therapy of this patient.

The patient had been monitored for 3 years and is still being followed and exhibits no claudication that limits his daily life. The follow-up CTA performed 2 years after the initial event revealed sustained occluded PA with prominent collaterals to the PTA from the incomplete SFA and only a slight enlargement of the PSAA to 24 mm.

## Discussion

Only a few studies have suggested a classification system for PSA. Two of the most well-liked categorization schemes are the Pillet–Gauffre (6, 7) and Ahn–Min (4) classifications. Both classification systems categorized PSA based on the completeness of PSA and SFA alongside the origin of PSA. The previously created Pillet–Gauffre classification was expanded by the Ahn–Min classification to include the presence or absence of aneurysmal degeneration, and it guides treatment methods based on the PSA class (Table 1).

The most common aspect of PSA is the anatomy with complete PSA and incomplete SFA, defined as a type 2a lesion in the Pillet–Gauff classification and class III in the Ahn–Min classification (4). Both our cases were classified as type 2a and class III lesions by both classifications, respectively. Conventional and recommended management for such lesions alongside limb ischemia comprises bypass surgery with the ligation of PA proximal to distal anastomosis (4). Additionally, embolization or surgical ligation of the aneurysm has been suggested for patients with PSAA (4). However, the collaterals from the incomplete SFA or DFA were not considered in these classifications when determining the treatment modality. In our cases, we performed an isolated thromboembolectomy of PA without bypass surgery in the first patient owing to adequate collaterals from the DFA and conservative management with anticoagulation and cilostazol in the second patient because of acceptable collaterals from the incomplete SFA. These two patients have been followed for 56 and 36 months without significant and lifestyle-limiting symptoms. Therefore, even though bypass surgery with PA ligation proximal to distal anastomosis may be the standard therapy, the approach should be tailored according to the anatomy and collateral flows of the patients.

The management of PSAA in these patients should also be considered. To the best of our knowledge, there is no definite threshold for the management of PSAA; however, in the presence of PSAA alongside distal thromboembolic events, especially with mural thrombus, the possibility of recurrent thromboembolism is inevitable following thrombectomy or thrombolysis, and treatment should be followed accordingly. However, we did not treat PSAA because the distal circulation was maintained *via* the collaterals from the DFA and SFA and not the PSA. Therefore, recurrent embolic events did not limit distal circulation in our cases. Furthermore, during the follow-up period, the PSAA of the first patient spontaneously occluded, and the aneurysm of the second patient demonstrated a slight increase in diameter without symptom development. Notably, a previous study has reported the growth of thrombosed PSAA with symptom aggravation following bypass surgery for limb ischemia in a patient with PSA (8). Hence, serial follow-up of PSAA is mandatory and specific guidelines for treating PSAA are warranted.

Owing to the recent advancement of endovascular devices and techniques, the endovascular management of PSAA and its associated complications has become an additional armamentarium for PSA management. The endovascular management of PSA can be divided into thrombolysis or pharmacomechanical thrombectomy for thromboembolic occlusion and stent-graft placement or coil embolization of PSAA. The CTA revealed type 2a PSA in our patients, signifying complete PSA and incomplete SFA. In contrast to the management of type 1 PSA with complete PSA and SFA, distal embolization should be managed through the PSA system in the endovascular approach. However, after managing thromboembolic occlusion, PSAA should be accessed as it can cause recurrent embolization or aneurysmal dilatation (8, 9). There have been some reports of successful distal embolization management through the PSA using the contralateral femoral or brachial approach (10). Recently, stent-graft placement for managing PSAA has garnered popularity, replacing conventional treatment (5). However, there are technical limitations to endovascular stent-graft repair, and its durability and long-term results have yet to be reported (11).

In summary, we described two patients with typical type 2a PSA according to the Pillet–Gauffre classification and class IIIa PSA according to the Ahn–Min classification. In both patients, bypass surgery was not performed despite distal embolization; however, distal circulation was preserved *via* the collaterals from the DFA and SFA without the additional risk of recurrent distal embolization. The current PSA classification systems only consider the completeness of the PSA and SFA and guide the treatment modality according to the classification, such as distal bypass surgery with ligation above the distal anastomosis of the bypass graft alongside endovascular or surgical treatment of PSAA, if present. However, the collaterals from the incomplete SFA and DFA are important for deciding the treatment modality; thus, carefully examining the CTA and developing a tailored approach is critical for managing PSA.

## Data Availability

The raw data supporting the conclusions of this article will be made available by the authors, without undue reservation.
